# Anticancer Activity and Mechanism of Xanthohumol: A Prenylated Flavonoid From Hops (*Humulus lupulus* L.)

**DOI:** 10.3389/fphar.2018.00530

**Published:** 2018-05-22

**Authors:** Chuan-Hao Jiang, Tao-Li Sun, Da-Xiong Xiang, Shan-Shan Wei, Wen-Qun Li

**Affiliations:** ^1^Department of Laboratory Medicine, The Second Xiangya Hospital, Central South University, Changsha, China; ^2^Key Laboratory Breeding Base of Hu’nan Oriented Fundamental and Applied Research of Innovative Pharmaceutics, College of Pharmacy, Changsha Medical University, Changsha, China; ^3^Department of Pharmacy, The Second Xiangya Hospital, Central South University, Changsha, China; ^4^Institute of Clinical Pharmacy, Central South University, Changsha, China; ^5^Hunan Provincial Engineering Research Center of Translational Medicine and Innovative Drug, Changsha, China

**Keywords:** xanthohumol, hops, phytochemical, anticancer, molecular mechanism

## Abstract

It has been observed that many phytochemicals, frequently present in foods or beverages, show potent chemopreventive or therapeutic properties that selectively affect cancer cells. Numerous studies have demonstrated the anticancer activity of xanthohumol (Xn), a prenylated flavonoid isolated from hops (*Humulus lupulus* L.), with a concentration up to 0.96 mg/L in beer. This review aims to summarize the existing studies focusing on the anticancer activity of Xn and its effects on key signaling molecules. Furthermore, the limitations of current studies and challenges for the clinical use of Xn are discussed.

## Introduction

**FIGURE 1:**
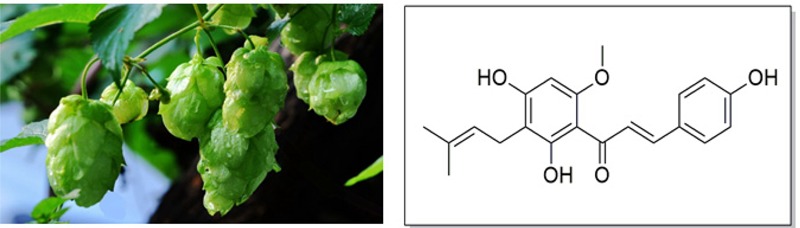
Hops (*Humulus lupulus* L.) and structure of Xn.

Hops (*Humulus lupulus* L.), a principal raw material of beer, have been widely used throughout the world in the brewing industry. It acts as a preservative to gives beer the unique aroma and flavor (McAdam et al., 2013; Dostalek et al., 2017). In addition, hops have been used as a medicinal plant for a long history owing to its richness in a variety of phenolic compounds (Zanoli and Zavatti, 2008). The dried hops contain 4–14% polyphenols, mainly phenolic acids, prenylated chalcones, flavonoids, catechins, and proanthocyanidins (Nikolic and van Breemen, 2013). Xanthohumol (Xn; 3′-[3,3-dimethylallyl]-2′,4′,4-trihydroxy-6′-methoxychalcone), the most abundant prenylated flavonoid with 0.1–1% of dry weight in hops, can be isolated from the female inflorescences as shown in **Figure 1**. It is also a constituent of beer, a major dietary source of prenylated flavonoids, where it has been found at concentrations up to 0.96 mg/L (1.95 μM) (Chen et al., 2012).

The structure of Xn was firstly identified by Verzele et al. (1957). However, the beneficial pharmacological properties of Xn were not appreciated until 1990s, including antioxidant, anti-inflammatory, antibacterial, antiviral, antifungal, and antiplasmodial activity (Liu et al., 2015). In recent years, increasing evidences suggested the anticancer activity of Xn against NSCLC, leukemia, HCC, breast cancer, PCa, CCA, glioblastoma, pancreatic cancer, colon cancer, cervical cancer, melanoma, thyroid cancer, laryngeal squamous cell carcinoma (LSCC), and ovarian cancer.

Present review firstly summarized the present knowledge of potential chemopreventive or therapeutic effects of Xn. Initially, we described the current evidence on anticancer activity of Xn including both *in vitro* and *in vivo* studies depending on various organs of the body, meanwhile the underlying mechanisms in each cancer type were discussed. Subsequently, the shortcomings of these studies were summarized, and which areas deserved to be further investigated. Additionally, the safety and imitations for clinical applications were presented. This review would contribute to the proceed for anticancer study of Xn, on the other hand, provide a reference for development of anticancer drugs.

## Respiratory System Cancers

### Non-small Cell Lung Cancer

Almost 1.6 million people are affected by lung cancer, and its 5-year survival rate is as low as 15% (Chen et al., 2014). Lung cancer mainly consists of two categories by histological classification: NSCLC and small cell lung cancer. However, the NSCLC patients account for about 85% of lung cancer patients per year (Yang and Lin, 2016).

NSCLC is classified as adenocarcinoma and squamous cell carcinoma (Kuribayashi et al., 2016). Most studies on anti-NSCLC effect of Xn are mainly focused on adenocarcinoma. Xn showed greater anti-proliferative activity against NSCLC adenocarcinoma cells A549 than H1563. The A549 cells appeared to have a high basal level of phosphorylated ERK1/2. Data suggested that the blockade of the Ras/Raf/ERK cascade was highly beneficial, especially when cancer cells growth was heavily dependent on MEK/ERK activity. Xn treatment caused concentration-dependent decrease of ERK1/2 phosphorylation in A549 cells. Xn also significantly reduced the phosphorylation of p90RSK and CREB, the downstream ERK1/2 substrates. The CREB is a well-known transcription factor whose active form binds to certain DNA sequences called cAMP response elements (CRE) and is responsible for the regulation of several target genes including cell cycle regulative gene (cyclin A, cyclin D1). As such, Xn treatment of A549 cells induced the up-regulation of cell cycle regulators p53 and p21 as well as down-regulation of cyclin D1 (Slawinska-Brych et al., 2016).

Promoting ROS over-production may be another important mechanism of how Xn induces apoptosis in A549 cells. Mitochondria are subcellular organelles whose primary function is to produce ATP through oxidative phosphorylation (OXPHOS). By dissecting the multiple steps of mitochondria OXPHOS with extracellular flux analysis, Zhang et al. (2015) found that Xn specifically inhibited the activity of complex I, and had little or no effects on complexes II, III, and IV. Inhibition of complex I (NADH-ubiquinone oxidoreductase) by Xn resulted in ROS over-production, which ultimately induced the apoptosis of A549 cells.

Summarized above the mentioned, Xn suppressed the ERK1/2 phosphorylation and promoted ROS over-production, ERK1/2 and ROS may be the target of Xn in NSCLC. However, the overexpression of ERK1/2 or ROS scavenger was not further used upon Xn treatment. Therefore, present studies were insufficient to confirm that ERK1/2 and ROS mediated the proliferation inhibition and apoptosis induction of Xn in NSCLC. Moreover, with the exception of *in vitro* studies, the anticancer activity of Xn in NSCLC should be verified through *in vivo* studies.

## Lymphatic Hematopoietic Cancers

### Leukemia

As the most frequent type of childhood cancer, ALL occurs particularly in children between the ages of 2 and 5 years. Currently, drug resistance during chemotherapy and the insurgence of neurological disorders emerge as the major obstacles for ALL cancer treatment (Cuvertino et al., 2017).

It has been reported that Xn can induce growth arrest and apoptosis in B-ALL cells. Moreover, Xn preserved equal cytotoxicity in adriamycin resistant ALL cells L1210, while long-term exposure of Xn to ALL cells improved sensitivity to chemotherapeutic drugs. These results suggested that Xn can effectively overcome the drug resistance of ALL, and promoted the sensitivity of chemotherapeutic drugs. In ALL-like xenograft mouse model, administration of Xn (50 μg/mouse/day in 200 μL PBS) significantly delayed the insurgence of neurological disorders, led to the increase of animal life span. This increased life expectancy was linked to a reduced ability of leukemic cells to infiltrate the CNS, as a delay of neurological symptoms was observed. Interestingly, Xn did not induce resistance but drug adaptation, characterized by the down-regulation of FAK, AKT, and NF-κB activities rendering cells less invasive and more susceptible to cytotoxic drugs (Benelli et al., 2012). Although the antileukemic activity of Xn both *in vitro* and *in vivo* have been investigated, positive drug group needs to be added in further studies, which can evaluated the anti-leukemic activity of Xn accurately and effectively.

In addition to ALL, Xn induced the apoptosis of chronic myeloid leukemia cells KBM-5, which attributed to the decrease of NF-κB activation via modifying IKK and p65 (Harikumar et al., 2009). It has been reported that PI3K/Akt, NF-κB, etc. signaling pathways can be activated by the oncogenic Bcr-Abl tyrosine kinase in Bcr-Abl (+) myeloid leukemia cells, eventually led to proliferation, transformation, and apoptosis resistance (Vakana and Platanias, 2011). Xn strongly inhibited Bcr-Abl expression in K562, a Bcr-Abl (+) myeloid leukemia cells. Unfortunately, the effects of Xn on the PI3K/Akt, NF-κB, etc. signaling pathways were not determined in K562, led to the precise molecular mechanism of Xn on B-CLL cells remained unknown (Monteghirfo et al., 2008).

## Digestive System Cancers

### Hepatocellular Carcinoma

The 5-year survival rate of HCC patients is 50–70% (Xie et al., 2015). Currently, liver transplantation is the only effective curative modality. However, surgery is often non-efficacious due to the potential metastasis of HCC. Therefore, there is an urgent need to find targeted anticancer agents with less toxic profiles for HCC patients (Thein et al., 2017). Kunnimalaiyaan et al. (2015a) found that treatment with Xn (5 μM or higher) for 4 days suppressed the cells viability, confluency, and colony forming ability of HCC cells (Huh-7, HepG2, Hep3B, and SK-Hep-1), which was indicated by increased expression of pro-apoptotic proteins (cleaved caspase-3 and c-PARP) and reduced expression of anti-apoptotic proteins (cyclin D1, survivin, and Mcl-1). Notch signaling pathway was involved in the anti-HCC effect of Xn, as evidenced by the decreased expression of Notch1 and HES-1 proteins (Kunnimalaiyaan et al., 2015a), and the anti-proliferative effect of Xn in HCC can be reversed by ectopic Notch1 expression (Kunnimalaiyaan et al., 2015a). Conversely, another study reported that Xn exhibited neither cytotoxic nor genotoxic to the HepG2 cells at concentrations up to 10 μM for 24 h (Plazar et al., 2007), which was consistent with the finding of Ho et al. (2008). They found the IC_50_ values of Xn for HA22T/VGH and Hep3B were 108 and 166 μM, respectively (Ho et al., 2008). Through comparative analysis, the inconsistent results might be due to the different treatment time (4 days vs. 24 h).

In addition to the growth suppression and apoptosis induction, a significant protective effect of Xn against pro-carcinogens induced DNA damage was observed at concentrations as low as 10 nM (Plazar et al., 2007). Moreover, Xn showed no effect on primary human hepatocytes viability at 100 μM or higher, while suppressed proliferation and migration of HCC cells (HepG2 and Huh7) at 25 μM via inhibiting NF-κB activity and IL-8 expression (Dorn et al., 2010). This finding suggested that Xn may be a safe and effective agent targeted cancer cells for HCC treatment, which needed to be further verified *in vivo*.

### Cholangiocarcinoma

As the first most common primary biliary malignancy and the second most common primary hepatic malignancy, CCA accounts for 3% of gastrointestinal tumors. Surgical restriction is the best treatment regimen for CCA. However, not all CCA patients are good candidates for curative surgery and complete surgical restriction is often followed by local recurrence with unsatisfactory 5-year survival rate (Rizvi and Gores, 2013). It has been reported that administration of Xn orally in drinking water to CCA bearing nude mice reduced tumor growth. Tumor tissues from Xn-treated mice exhibited suppressed tumor growth and increased apoptosis as demonstrated by decreased Ki67 positive cells and increased TUNEL positive cells (Dokduang et al., 2016). However, it was difficult to guarantee the identity among individuals through administration of Xn orally in drinking water. Because of the poor solubility in water, Xn can be dispersed in suspending agent (e.g., sodium carboxymethyl cellulose), and intragastric administrated to CCA mice.

Additionally, the STAT3 activation was reduced in Xn-treated mice. *In vitro* study also found that Xn significantly inhibited cell proliferation in both KKU-M139 and KKU-M214 through the suppression of STAT3 activation. In addition, Akt and NF-κB p65 nuclear translocation activity was decreased after treatment with Xn in both the IL-6-induced CCA cells and the CCA inoculated mice (Dokduang et al., 2016). In view of the fact that STAT3 activation was regulated by Akt/NF-κB signaling pathway, the possible mechanisms by which Xn suppressed STAT3 activation in CCA may be due to Akt/NF-κB signaling inhibition.

### Pancreatic Cancer

Pancreatic cancer is now the third leading cause of cancer-related death in the United States, with pancreatic ductal adenocarcinoma (PDAC) representing the majority of these cases. Despite a steady increase in survival for most cancers over the decades, the 5-year survival of PDAC remains essentially unchanged at 8% (Siegel et al., 2017). Therefore, there is an urgent need to develop new therapeutic strategies with low toxicities to treat PC. Xn treatment was found to arrest cell cycle and induce apoptosis in pancreatic cancer cells (PANC-1, BxPC-3) via decreasing STAT3 phosphorylation and its downstream targeted genes expression, such as cyclin D1, survivin, and Bcl-xL. The further *in vivo* study demonstrated that Xn can suppress the pancreatic cancer growth in a xenograft nude mouse model. However, the STAT3 phosphorylation and its downstream targeted genes expression in tumor tissue were not determined, so that STAT3 signaling pathway whether acted as the key molecular target of Xn in pancreatic cancer pending further study (Jiang et al., 2015). Another study suggested that Xn reduced the proliferation of pancreatic cancer cells (AsPC-1, L3.6pl, PANC-1, MiaPaCa-2, 512, and 651) in a dose and time dependent manner. The growth suppression effect of Xn was due to increased apoptosis via the inhibition of the Notch1 signaling pathway, as evidenced by reduction in Notch1, HES-1, and survivin both at mRNA as well as protein levels (Kunnimalaiyaan et al., 2015b). The relationship between STAT3 and Notch1 signaling pathway has been reported in multiply kinds of cancer, either coordination or interaction (LeComte and Spees, 2016). The detail relationship in Xn-treated pancreatic cancer required further investigation.

### Colon Cancer

Colorectal cancer is currently the second most common cancer in women and the third most common in men, with 1.4 million new cases annually. The global burden of colorectal cancer is expected to increase by 60% to more than 2.2 million new cases and 1.1 million deaths by 2030 (Ouban, 2018). Phytochemicals in foods or traditional medicines have become one of the multi-therapy approach choices for cancer treatment. Six prenylated flavonoids isolated from hops were tested for their anti-proliferative activity in colon cancer cells (HT-29). Result showed that Xn, DX (dehydrocycloxanthohumol) and IX (isoxanthohumol) dose-dependently decreased HT-29 proliferation (0.1–100 μM) (Miranda et al., 1999). Among them, Xn exhibited the best cytotoxic activity. The other five prenylated flavonoids are the Xn structural analogs, therefore, their structure–activity relationship may provide reference for further structural modification. In 40-16 human colon cancer cells, Xn significantly reduced its proliferation with IC_50_ values of 4.1, 3.6, and 2.6 μM for 24, 48, and 72 h, respectively (Pan et al., 2005). The cytotoxicity of Xn on HCT-15 was also evaluated. Xn showed potent cytotoxicity against HCT-15 with IC_50_ values of 3.6 μM after 24 h of treatment. Moreover, Xn clearly decreased the expression of the drug efflux genes including ABCB1 (MDR1), ABCC1 (MRP1), ABCC2 (MRP2), and ABCC3 (MRP3) (Lee et al., 2007), which suggested that Xn might be used in combination with other anticancer chemotherapeutic agents to reduce the drug resistance by inhibiting the efflux drug transporters.

## Genitourinary Organ Cancers

### Breast Cancer

Because of the crucial roles of progesterone receptor (PR), estrogen receptor (ER) and human epidermal growth factor receptor 2 (HER2) in breast cancer carcinogenesis, it is classified based on their expression: (1) ER^+^, estrogen receptor positive; (2) HER2^+^, overexpressing human epidermal growth factor receptor 2, which can be ER^+^ or ER^-^; (3) TNBCs, triple negative subtype that do not express estrogen, progesterone, and HER2 receptors. Up to date, effective therapeutics for TNBCs has not been found (Jitariu et al., 2017).

Hs578T and MDA-MB-231 have been used as TNBCs extensively in *in vitro* studies (Bassey-Archibong et al., 2016). Treatment with Xn for 24 h inhibited the growth of MDA-MB-231 and Hs578T with IC_50_ values of 6.7 and 4.78 μM, respectively. Xn also inhibited the invasive phenotype of Hs578T and MDA-MB-231 (Kim et al., 2013). The caspase- and mitochondria-dependent apoptotic pathway mediated the apoptosis-induced effect of Xn on MDA-MB-231 cells (Yoo et al., 2014). In addition, it was reported that the exposure of Xn (10 μM, 48 h) to MCF-7 cells can inhibit cell proliferation, accompanied with a significant reduction in alkaline phosphatase (ALP) activity (Guerreiro et al., 2007). The abnormal expression of ALP isoenzymes, identified as a prognostic biomarker, was found in multiple malignant tissues (Wada et al., 2001). *In vivo* study indicated that oral administration of Xn to MCF-7 bearing nude mice caused central necrosis within tumor tissues, focal proliferation areas, reduced inflammatory cell number, decreased microvessel density, and increased percentage of apoptotic cells (Monteiro et al., 2008). It has been reported that MCF-7 was the ER^+^ breast cancer cells, while MDA-MB-231 belongs to TNBCs. Compared with the MCF-7, MDA-MB-231 exhibits stronger metastasis ability and more susceptibility to tumorigenicity in nude mice. Further *in vivo* studies can be conducted in MDA-MB-231 bearing nude mice, to evaluate the difference between ER^+^ and TNBCs subtype after Xn treatment, which may decide the direction of clinical studies.

The further *in vitro* study focused to MCF-7/ADR cell line, which is resistant to many kinds of anticancer drugs, to evaluate the sensitivity of this cell line to Xn. Results showed that Xn dose-dependently induced apoptosis, decreased cell viability and arrested the cell cycle of MCF-7/ADR cells. When detecting the apoptosis related proteins, including Bax, Bcl-2, procaspase-3, and cleaved-PARP, the increased level of γ-H2AX was observed. This finding indicated the cytotoxic effect of Xn possibly had relationship with its genotoxic effect. Moreover, Xn could down-regulate the cancer stemness characters in MCF-7/ADR cells, as evidenced by the decreased SP percentage in MCF-7/ADR cells upon Xn treatment. SP cells (breast cancer stem-like cells) are characterized by their high clonogenicity, increased migration, and resistance to the fluorescence dyes. Therefore, agents that can reduce the SP phenotype are currently in vogue in cancer therapeutics (Liu M. et al., 2016). Moreover, the radio- and chemo-sensitizing studies of Xn on MCF-7/ADR cells showed that Xn can recover the sensitivity of MCF-7/ADR cells to radiation and adriamycin therapies, which inhibited EGFR, MDR1, and STAT3 expression. These results suggested that Xn may be a potent chemo- and radio-sensitizer, which deserved to be further verified in animal or human (Kang et al., 2013).

### Prostate Cancer

The PCa is the most common death in men worldwide (Cao et al., 2016). PCa cells can be classified as AR^+^ (hormone-sensitive) subtype and AR^-^ (hormone-refractory) subtype. LNCaP is the typical AR^+^ cells, while PC-3 and DU145 belong to AR^-^ cells. These cells were all highly sensitive to Xn at a concentration range of 20–40 μM, The primary mechanism of action of Xn was to induce cell apoptosis as demonstrated by the binding of annexin V-FITC, cleavage of PARP-1, activation of procaspase-3, -8, and -9, mitochondrial depolarization, and release of cytochrome *c* from mitochondria. Induction of apoptosis by Xn was associated with the inhibition of prosurvival Akt, NF-κB, and p-mTOR as well as NF-κB-regulated anti-apoptotic Bcl-2 and survivin (Deeb et al., 2010). In malignant PCa cells PC3 and non-tumorigenic prostate hyperplasia cells BPH-1, Xn dose-dependently decreased cells viability (2.5–20 μM) and increased early and late apoptotic cells (Colgate et al., 2007). Interestingly, BPH-1 cells were more sensitive to growth inhibitory and pro-apoptotic effects of Xn than cancerous PC-3 cells. Thus, the more potential efficacy of Xn may be laid in precancerosis or prostate hyperplasia, suggesting that Xn served as a promising chemopreventive agent in PCa.

The *in vivo* study was conducted in the TRAMP transgenic mice, a PCa animal model. Oral administration of Xn did not prevent the initiation of carcinogenesis but decreased the average urogenital tract weight, inhibited the growth of poorly differentiated prostate carcinoma and delayed the tumor progression. Further *in vitro* study indicated that Xn can inhibit proliferation and reduce cell migration and invasion of DU145 and PC3 at low micromolar concentration, these effects were associated with down-regulated FAK and AKT phosphorylation, as well as increased ROS (Vene et al., 2012). TRAMP transgenic mice act as a spontaneous PCa model, the degree of malignancy is increased over time. In this study, Xn was administrated by gavage to TRAMP mice beginning at 4 weeks and continuing until the animals were 24 weeks old, therefore, which was considered to be a preventive mode. The results indicated that Xn was a promising chemopreventive agent for PCa indeed. Further study can be conducted in a therapeutic model.

TRAIL, an endogenous ligand, plays a crucial role in anti-tumor immunity and immune surveillance (Chen et al., 2017), while LNCaP PCa cells are resistant to TRAIL-induced apoptosis. It should be noted that Xn increased sensitivity of LNCaP to TRAIL, which maybe another important mechanism of Xn-induced PCa cells apoptosis (Klosek et al., 2016).

### Cervical Cancer

Cervical cancer constitutes a leading cause of morbidity and cancer deaths in women throughout the world. Approximately two-thirds of the patients are diagnosed with locally advanced cervical cancer, showing disappointing survival rates (Marquina et al., 2018). Thus, an effective and safe therapy for cervical cancer is urgently needed. Yong et al. (2015) found the induction of apoptosis by Xn on Ca Ski cervical cancer cell line. Additionally, Xn mediated S phase arrest in cell cycle analysis and increased activities of caspase-3, -8, and -9. On the other hand, the expression of cleaved PARP, p53, and AIF were increased, while Bcl-2 and XIAP were decreased in a dose-dependent manner. These findings indicate that Xn-induced apoptosis might involve intrinsic and extrinsic apoptotic pathways (Yong and Abd Malek, 2015). Obviously, the anticancer activity of Xn against cervical cancer was only investigated in Ca Ski cells *in vitro* to date. Therefore, more types of cervical cancer cells (e.g., Hela and SiHa) and *in vivo* model may be supposed to evaluate the anticancer activity of Xn against cervical cancer.

### Ovarian Cancer

Ovarian cancer is the fifth most common cancer in the United States and is the deadliest gynecologic malignancy. Due to the difficulty of early detection, most cases of ovarian cancer are stage III or IV when discovered resulting in only a 15–20% cure rate (Musella et al., 2018). After exposure to A-2780 ovarian cancer cells for 2 days and 4 days, Xn induced highly cytotoxicity with IC_50_ values of 0.52 and 5.2 μM, respectively (Miranda et al., 1999). Significant growth inhibition and down-regulation of Notch1 transcription and protein expression were found following Xn treatment in SKOV3 and OVCAR3 cells (Drenzek et al., 2011). However, it has been reported that metastasis is the major cause of morbidity and mortality in patients with ovarian cancer, especially the epithelial ovarian cancer (Weidle et al., 2016). Many efforts can be made to evaluate the metastasis ability upon Xn treatment, in addition to cytotoxicity and proliferation.

## Head and Neck Cancers

### Glioblastoma

Glioblastoma multiforme (GBM), a grade IV malignant glioma, is the most aggressive primary brain tumor with a poor prognosis in adults. With the gradually increasing drug resistance with TMZ treatment, the first line chemotherapeutic drug in clinical gliomas therapy, innovative replacement or adjuvant drugs for glioblastomas are urgently needed (Reitman et al., 2018). Xn decreased viability and induced apoptosis of T98G cells in a time- and concentration-dependent manner. These effects involved activation of caspase-3, -9, and PARP cleavage as well as increase of intracellular ROS production (Festa et al., 2011). Chen et al. (2016) found that Xn significantly induced the apoptosis and reduced cell viability in U87 cells. In view of the key roles of miRNAs in the development of glioblastoma, the microarray analysis was used to identify the abnormal expressed miRNAs under Xn treatment. Results showed that miR-204-3p was the most up-regulated miRNA. The ERK/c-Fos pathway mediated the effect of Xn on miR-204-3p expression. Furthermore, miR-204-3p targeting IGFBP2/AKT/Bcl2 pathway regulated U87 cells death (Chen et al., 2016). The miRNAs serves as the important member of ncRNAs. The ncRNAs are a heterogeneous class of transcribed RNA molecules. Several studies have shown that ncRNAs dysregulation is a common central event occurring in multiple cancers and has the potential of being therapy targets (Wangyang et al., 2018). Recently, increased evidences suggested the ncRNAs mediated the anticancer activity of phytochemical constituents (Gulei et al., 2017). The anticancer activity of Xn whether also involved other ncRNAs, e.g., lncRNAs and circRNAs, remains unknown.

### Thyroid Cancer

There has been a worldwide thyroid epidemic with 470,000 women and 90,000 men being diagnosed during the last two decades. Though the disease-specific mortality of thyroid cancer is low, unwarranted or inadequate surgery is associated with increased morbidity making proper management important (D’Cruz et al., 2018). The promotion of Xn on I^-^ uptake of FRTL-5 thyrocytes suggested that Xn might be an interesting candidate for increasing the sensitivity of radiotherapy on thyroid cancer (Radovic et al., 2005). Furthermore, treatment of medullary thyroid cancer cells MTC with Xn dose-dependently inhibited proliferation and down-regulated ASCL1, a possible malignant phenotype in thyroid cancer (Cook et al., 2010). However, the inhibition rate reminds lower than 50% after 4 days of treatment with 30 μM Xn. Therefore, whether Xn exhibits the potent anticancer activity in thyroid cancer, similarly to other cancers, needs to be further verified.

### Laryngeal Squamous Cell Carcinoma

Laryngeal squamous cell carcinoma is the most common malignancy of the head and neck worldwide. The 5-year survival rates for laryngeal cancer patients with regional and metastatic LSCC significantly decreased within last three decades, irrespective of the therapy applied (Cosetti et al., 2008). Therefore, there is a need for discovery of novel, more effective therapeutic agents. It has been reported that Xn treatment reduced the cell viability of LSCC cells (RK33 and RK45), but had very low or no toxicity in normal cells (rat oligodendroglia-derived cells, OLN-93 and human skin fibroblasts, HSF) (Slawinska-Brych et al., 2015). Moreover, Xn exhibited potent cytotoxicity in SCC4 cells, which may be associated with suppression on Mcl-1 and Bcl-2 expression, as well as PARP, p53, and AIF activation (Li et al., 2016). Although changes in these protein expressions or activities have been observed upon Xn treatment, lack of knockdown or overexpression methods to further determine the molecular target of Xn in LSCC.

## Other Cancers

### Melanoma

It is known that melanogenesis can be induced by several extracellular stimuli except ultraviolet (UV) light, such as isobutylmethylxanthine (IBMX) (Siegel et al., 2017). Xn dose-dependently (0.5–10 μM) inhibited IBMX-induced melanogenesis in B16 melanoma cells, which was associated with the reduced tyrosinase enzyme activity (Koo et al., 2008). Tyrosinase, a bifunctional enzyme, was responsible for melanin production (Hearing and Jimenez, 1987). In another melanoma cells SK-MEL-2, Xn showed potent cytotoxicity by inhibiting the activity of DNA topoisomerase I (Topo I), suggesting Xn may be a novel Topo I inhibitor (Lee et al., 2007). Topo I is a nuclear enzyme engaged in adjustment of DNA topological structure during cell cycle. Inhibition of this enzyme results in DNA strand breaks, ultimately leads to proliferation inhibition and apoptosis induction. Consequently, Topo I has a great potential as a target for the treatment various cancers. For instance, 10-hydroxycamptothecin, a Topo I inhibitor, has been used as a broad-spectrum anticancer drug in clinical practice (Gokduman, 2016). Thus, the broad-spectrum anticancer activity of Xn might be attributed to its inhibition on Topo I activity.

## Discussion and Perspective

### Common Diet With Cancer Preventive and Therapy Phytochemicals

Cancer is a complex multifactorial disease, in which normal cells are transformed into malignant cells acquiring several properties including enhanced proliferation and apoptosis-resistance (Wang et al., 2014; Ouyang et al., 2016). Due to the mechanisms of carcinogenesis and cancer development are still unclear, preventive intervention is becoming scientifically practical for treating cancers (Kelloff et al., 1994; Sporn and Suh, 2002). Several studies have demonstrated that diet represents a new strategy in cancer management, that means prevention starts from what we drink and what we eat (Rossi et al., 2014). Dietary factors, found in solid foods and beverages, contribute to the prevention of approximately 30% of cancers in industrialized countries, and diet acts as the second preventive approach in cancer (Rodriguez-Casado, 2016). It has been observed that numerous phytochemical components, frequently present in beverages, show potent chemopreventive or therapeutic properties that selectively affects cancer cells (Wu et al., 2017). For instance, EGCG, the major class of flavonoids isolated from green tea (Schramm, 2013), showed a chemopreventive effect on a variety of human cancers *in vitro* and *in vivo*, and even in recent clinical trials (Du et al., 2012; Zhao et al., 2015, 2016). The well-known stilbenoid resveratrol, found in grape and red wine, exerted anticancer activity in various malignant cancers (Liu et al., 2007; Zhang et al., 2014; Pan et al., 2017). Xn was the most abundant flavonoid isolated from the hop plant (*H. lupulus* L.), with a concentration of up to 0.96 mg/L in most beers (Stevens et al., 1999), and reach 3.5 mg/L in dark beer through using colored malt (Magalhães et al., 2008). Over almost 30 years, increasing evidences have emerged concerning the anticancer activity of Xn.

### Anticancer Activity of Xn

The data from the *in vitro* studies indicated that Xn was able to inhibit both carcinogenesis and metastasis. As summarized in **Table 1**, exposure of cancer cells to Xn inhibited the proliferation, migration, and invasion, as well as induced apoptosis and cell cycle arrest. The range of anticancer activity covered multiple parts of body including respiratory system, digestive system, genitourinary system, and more. However, most of the *in vitro* studies were not compared with the standards of care, so that it would not provide a good reference for further *in vivo* animal and clinical studies.

**Table 1:** Anticancer activity of Xn *in vitro*.

Cancer types		Cell lines	Dose or IC_50_ (μM)	Duration (h)	Finding	Mechanism	Reference
Respiratory system cancers	NSCLC	A549	5–25	48	↓ Cell viability ↓ Proliferation ↑ Apoptosis	↓ ERK1/2 ↓ p90RSK kinases ↓ CREB ↑ p53 and p21 ↓ Cyclin D1 Cycle arrest (G1 phase) ↑ Caspase-3 activity	Slawinska-Brych et al., 2016
		A549	5–30	6, 24	↑ Apoptosis	↓ NADH dehydrogenase ↑ Glycolysis ↑ ROS	Zhang et al., 2015
		A549	14, 28, 42	24, 48, 72	↓ Proliferation ↑ Apoptosis	Cycle arrest (S phase) ↑ Caspase-3, -8, -9	Yong et al., 2015
Lymphatic hematopoietic cancers	Leukemia	Acute lymphoblastic leukemia L1210 and adriamycin-resistant L1210	2.5, 5, 10	48, 96	↓ Cell viability ↑ Apoptosis ↓ Migration ↓ Invasion	↓ FAK, ↓ AKT ↓ NF-κB signaling pathways	Benelli et al., 2012
		Chronic myeloid leukemia KBM-5	50	4	↑ Apoptosis ↓ Invasion	↓ IKK activity ↓ IκBα phosphorylation and degradation ↓ p65 nuclear translocation ↓ NF-κB controlled antiapoptotic genes (Bcl-xL, cIAP-1, cIAP-2, XIAP, survivin, and TRAF-2)	Harikumar et al., 2009
		Bcr-Abl (+) myeloid leukemia cells K562	2.5, 5, 10	24	↓ Cell viability ↑ Apoptosis ↓ Invasion ↓ Adhesion to endothelial cells	↓ Bcr-Abl ↑ p21 ↑ p53 ↓ MMP-2	Monteghirfo et al., 2008; Vakana and Platanias, 2011
Digestive system cancers	Hepatocellular carcinoma	Huh-7, HepG2, Hep3B, and SK-Hep-1	5	96	↓ Cell viability ↓ Colony forming ↓ Confluency ability	↓ Notch1 ↓ HES-1	Kunnimalaiyaan et al., 2015a
		HepG2	10	24	None	None	Plazar et al., 2007
		HA22T/VGH and Hep3B cells	IC_50_ = 108, 166	24	None	None	Ho et al., 2008
	Cholangiocarcinoma	KKU-M214, KKU-M139	20, 50	24, 48,72	↓ Cell growth	↓ STAT3 activation	Dokduang et al., 2016
	Pancreatic cancer	PANC-1, BxPC-3	5–100	48	↓ Cell viability ↓ Colony formation ↑ Apoptosis	↓ p-STAT3	Jiang et al., 2015
		AsPC-1, PANC-1, L3.6pl, MiaPaCa-2, 512, 651	10	48-96	↓ Proliferation	↓ Notch1 ↓ HES-1 ↓ Survivin	Kunnimalaiyaan et al., 2015b
	Colon cancer	HT-29	0.1–100	48, 72	↓ Cell viability	None	Miranda et al., 1999
		40-16 colon cancer cells	IC_50_ = 4.1, 3.6, 2.6	24, 48, 72	↓ Proliferation	None	Pan et al., 2005
		HCT-115	IC_50_ = 10.2	24	↓ Proliferation	↓ ABCB1 ↓ ABCC1, ABCC2, ABCC3	Lee et al., 2007
Genitourinary organ cancers	Breast cancer	MDA-MB-231 and Hs578T	IC_50_ = 6.7, 4.78	24	↓ Proliferation ↓ Cell invasion	None	Kim et al., 2013
		MDA-MB-231	10, 20	48	↓ Cell viability	↓ Bax ↑ Caspase-3, -9	Yoo et al., 2014
		MCF-7	10	48	↓ Proliferation	↓ ALP isoenzymes	Guerreiro et al., 2007
		Adriamycin-resistant MCF-7 (MCF-7/ADR)	10	24, 48	↓ Cell viability ↑ Apoptosis ↓ Stemness ↑ Chemo-sensitizing ↑ Radio-sensitizing	↑ γ-H2AX ↓ MDR1 ↓ EGFR ↓ STAT3	Kang et al., 2013; Liu M. et al., 2016
	Prostate cancer	Hormone-sensitive (AR^+^): LNCaP; hormone-refractory (AR^-^): PC-3 and DU145	20, 40	72	↓ Cell viability ↑ Apoptosis	↓ p-AKT ↓ NF-κB p65 ↓ p-mTOR ↓ Bcl-2 ↓ Survivin	Deeb et al., 2010
		Hormone-refractory (AR^-^): PC-3	2.5–20	48h	↓ Cell viability ↑ Apoptosis	↓ NF-κB activation	Colgate et al., 2007
		Hormone-refractory (AR^-^): PC-3 and DU145DU145, PC3	2.5, 5, 10	48, 96	↓ Proliferation ↓ Cell migration ↓ Cell invasion	↓ p-FAK ↓ p-AKT	Vene et al., 2012
	Cervical cancer	Ca Ski	IC_50_ = 59.96 ± 1.95, 34.01 ± 1.13, 20.08 ± 1.12	24, 48, 72	↓ Proliferation ↑ Apoptosis	↓ XIAP ↑ p53 ↑ Caspase-3, -8, -9 Cycle arrest (S phase)	Yong and Abd Malek, 2015
	Ovarian cancer	A-2780	IC_50_ = 0.52, 5.2	48, 96	↓ Proliferation	None	Miranda et al., 1999
		SKOV3, OVCAR3	10, 20, 30	96, 144	↓ Proliferation	↓ Notch1 ↑ p21 Cycle arrest (S and G2/M)	Drenzek et al., 2011
Head and neck cancers	Glioblastoma	U87 glioblastoma cells	25	24	↓ Cell viability ↑ Apoptosis	↑ ERK/c-Fos ↑ miR-204-3p ↓ IGFBP2/AKT/Bcl_2_	Chen et al., 2016
		T98G	20	24, 48	↓ Cell viability ↑ Apoptosis	↑ ROS ↑ p-ERK1/2 ↑ p-p38 ↑ Caspase-3, -9 ↑ PARP cleavage	Festa et al., 2011
	Thyroid cancer	Medullary thyroid cancer cells MTC	10, 20, 30	96	↓ Proliferation ↓ Malignant phenotype	↑ ERK1/2 phosphorylation	Cook et al., 2010
	Laryngeal squamous cell carcinoma	RK33 and RK45	IC_50_ = 12.3, 22.5	48	↓ Cell viability ↑ Apoptosis	↑ p53 and p21 ↓ Cyclin D1 ↓ ERK1/2 phosphorylation ↑ Caspase-3, -8, -9 Cycle arrest (G1 phase)	Slawinska-Brych et al., 2015
		SCC4	20, 30, 40	48	↓ Proliferation ↑ Apoptosis	↓ Bcl-2 ↓ Mcl-1 ↑ PARP ↑ p53 ↑ AIF	Li et al., 2016
Other cancers	Melanoma	B16	0.5, 1, 5, 10	48	↓ IBMX-induced melanogenesis	↓ Tyrosinase enzyme activity, protein and mRNA	Koo et al., 2008
		SK-MEL-2	IC_50_ = 14.4	24	↓ Proliferation	↓ DNA topoisomerase I	Lee et al., 2007

Among these kinds of cancer, the activity of Xn on breast cancer and leukemia were systematically studied *in vitro*. In addition to the inhibition on carcinogenesis and metastasis, Xn can increase the chemo- and radio-sensitizing of adriamycin-resistant MCF-7 cells (Kang et al., 2013), similar results were also observed in adriamycin-resistant ALL cells L1210 (Benelli et al., 2012). These findings indicated that Xn may work synergistically with the current traditional chemotherapy and radiotherapy treatments to decrease the doses that often result in toxicity and severe side-effects.

Apart from *in vitro* studies, the *in vivo* animal study showed that administration of Xn in drinking water to CCA bearing mice can reduce tumor growth (Dokduang et al., 2016). Intraperitoneal injecting Xn significantly delayed the insurgence of neurological disorders in ALL-like xenograft mouse model, led to the increase of animal life span (Benelli et al., 2012). Moreover, oral gavage of Xn to PCa mice decreased the average weight of the urogenital tract, inhibited the growth of poorly differentiated prostate carcinoma and delayed the advanced tumor progression (Vene et al., 2012). Unfortunately, only a handful of *in vivo* studies investigated the anticancer activity of Xn, no clinical studies have been done in humans. Therefore, future *in vivo* animal and human studies should determine the effective dose and best route of Xn administration and side effects related to chronic administration.

Throughout these studies mentioned above, a comprehensive study was carried out only in ALL, including *in vitro, in vivo*, and the drug resistance. Accordingly, anticancer activity of Xn on ALL can be priorities for future intensive research.

### Anticancer Mechanisms of Xn

**FIGURE 2:**
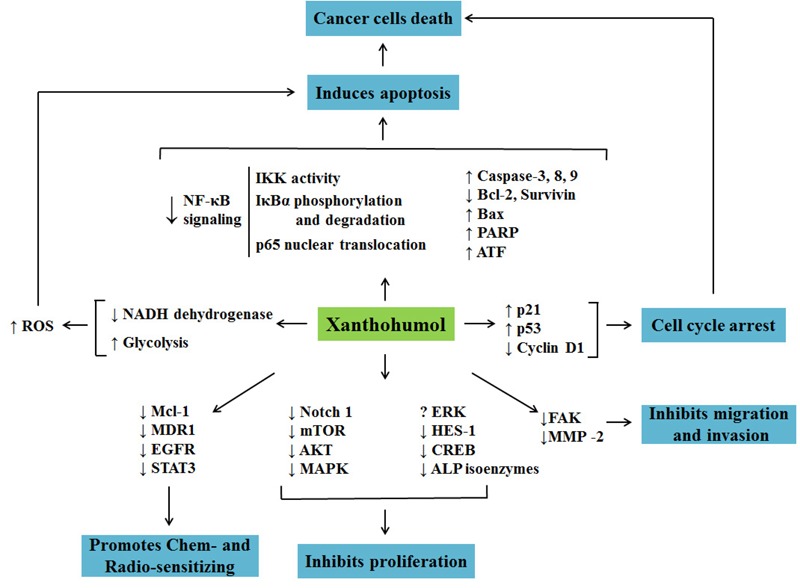
Cellular and molecular mechanisms involved in anticancer activity of Xn.

Recently, the focus for cancer treatment has been shifted toward finding chemicals/strategies that specifically target crucial signaling molecules/ pathways in cancer (Basak et al., 2017). As shown in **Table 1** and **Figure 2**, there were several lines of evidences suggesting the inhibition of Xn on Akt and NF-κB signaling pathways, which played key roles in the maintenance malignant phenotypes and the metastasis of cancer. The up-regulation of the pro-apoptotic proteins Bax, PARP, AIF, caspase-3, -8, -9 and down-regulation of anti-apoptotic protein Bcl-2 were involved in the Xn-induced cancer cells apoptosis, while the reduced Notch1, mTOR, STAT3, etc. mediated the inhibitive effect of Xn on cancer cells proliferation. Furthermore, Xn induced cell cycle arrest via regulating the expression of p53, p21, and cyclin D1. The down-regulation of FAK and MMP-2 expression was involved in the inhibition of Xn on migration and invasion of cancer cells. It is well known that MDR1, EGFR, and STAT3 are in significant associations with the multidrug resistant of cancer cells (Lee et al., 2011; Liu T. et al., 2016; Singh and Silakari, 2017). These proteins were down-regulated in Xn-treated MCF-7/ADR, suggesting that it was possible for Xn to act synergistically with the current traditional chemotherapy treatments and decrease the doses. Several studies presented conflicting results regarding the effect of Xn on ERK1/2 phosphorylation. For instance, Xn treatment increased ERK1/2 phosphorylation in thyroid cancer cells, while reduced ERK1/2 phosphorylation in larynx cancer cells. The reason may lie beyond the different effects of Xn on ERK1/2 phosphorylation was cell specific. Collectively, it can be easily noticed that Xn affects a series of key proteins associated with cancer cells proliferation, apoptosis, migration, invasion, and multidrug resistance. However, the exact molecular target of Xn remained unclear. Trace its root, lack of knockdown or overexpression methods to verify the aforementioned signaling molecules, and even no gene knockout animal models are used to evaluate the anticancer activity of Xn.

### Safety Studies for Xn Application

During the course of anticancer activity studies, the effect of Xn on normal cells was simultaneously investigated. Xn exhibited very low or no toxicity in normal cells including human lung fibroblast cells (MRC-5), primary human hepatocytes, oligodendroglia-derived cells (OLN-93), and human skin fibroblasts (Dorn et al., 2010; Slawinska-Brych et al., 2015; Yong et al., 2015). These findings suggested that Xn specifically targeted cancer cells, on the other hand, Xn may be a safe and effective agent. Similar results were obtained *in vivo*. Vanhoecke et al. (2005) reported the results of a 4-week safety study of Xn in mice. Daily administration of 23 mg/kg BW did not cause any noticeable sign of toxicity in bone marrow, liver, exocrine pancreas, kidneys, muscles, thyroid, and ovaries (Vanhoecke et al., 2005). Moreover, Hussong et al. (2005) investigated the sub-chronic toxicity of Xn in female SD rats at daily doses up to 1000 mg/kg BW for 4 weeks, which causes weak hepatotoxicity in female SD rats, but does not influence reproduction and the development of two generations of offspring when given at a daily dose of 100 mg/kg BW. However, all of these *in vivo* studies were conducted in normal animal. Ideally chemopreventive agents given at safe doses effectively affected the carcinogenic process without toxicity. In order to simultaneously confirm the safety and effectiveness, the toxicity to normal organs should be measured in tumor-bearing mice after treatment with Xn.

### Limitations for Clinical Applications of Xn

The current literatures available provide supporting evidence for the use of Xn as an anticancer agent, but there are several barriers from basic research to clinical practice. The low bioavailability is one of the important limitations (Venturelli et al., 2016). Female SD rats were treated with 1000 mg/kg BW, 80% of the applied Xn was excreted in feces and urine after stopping gavage for 48 h (Nookandeh et al., 2004). Avula et al. (2004) collected plasma, urine, and feces at varying time points and determined the Xn concentration, found that most of Xn were excreted in feces within 24 h of administration. These studies suggested the very low bioavailability of Xn after oral administration. In order to find out the underlying cause of low bioavailability, several pharmacokinetics studies in intestinal cells have been performed. A 70% of Xn added in the apical side of Caco-2 cells was found to accumulate inside the cells, and 93% of the intracellular Xn was localized in the cytosol and bound to cellular proteins, which may be the major factor responsible for poor oral bioavailability *in vivo* (Pang et al., 2007). Studies on the interaction of Xn with phosphatidylcholine membranes found that Xn inserted into lipid bilayers, thereby affected molecular organization and biophysical properties of the bilayer (Arczewska et al., 2013; Wesolowska et al., 2014). On this basis, the future studies on chemical structure modification and optimization of physical and chemical properties can focus on how to decrease specific binding of Xn to cytosolic proteins in intestinal epithelial cells and promote the rapid transport through the cell membrane, ultimately increase the bioavailability of Xn.

In addition to the low bioavailability, low extractive yield is another important factor restricting clinical application. Xn existed ubiquitously within hops plant in nature, which can be secreted as a part of the hop resin (Dresel et al., 2015). Originally, repeated chromatographic separations on silica gel were used to isolate and purify Xn. With the high-speed counter-current chromatography method being established, the extractive yield has been improved. However, it was still unable to meet the clinical needs (Chen et al., 2012). Therefore, a total synthesis method for Xn was further investigated (Khupse and Erhardt, 2007; Ellinwood et al., 2017). Unfortunately, the process was complicated and the overall yield is relatively low (10% overall yield from phloracetophenone after six steps) (Khupse and Erhardt, 2007). Learning from the development of Paclitaxel, further studies can focus on improvement of total synthesis yield. Paclitaxel in the market is known to be primarily obtained by this semi-synthetic route independent of isolation from plant (taxus chinensis)(Gueritte, 2001; Fu et al., 2009).

## Conclusion

This review summarized the current advances on Xn, a prenylated flavonoid from hops (*H. lupulus* L.) with potent anticancer activity to inhibit carcinogenesis and metastasis in many types of cancer, and additional properties that can be used as chemo- and radio-sensitizer. Multiple crucial signaling molecules and pathways were involved in the anticancer activity of Xn, such as Akt, NF-κB, ROS, ERK1/2, and so on. In view of these properties, Xn appears to be a good candidate in prevention and treatment for cancer and could be a potential weapon for new therapeutic strategies. However, more molecular biology techniques (e.g., knockdown and overexpression) should be applied in mechanism studies, more systematic *in vivo* studies are required to acquire a better understanding of the anticancer activity of Xn. The bioavailability and extraction yield remain to be improved in further clinical application.

## Author Contributions

C-HJ and T-LS reviewed the literature and wrote the article. D-XX contributed valuable opinions in accomplishing the article. S-SW contributed valuable opinions in the construction of infrastructure of the article. W-QL guided and supervised writing of the article.

## Conflict of Interest Statement

The authors declare that the research was conducted in the absence of any commercial or financial relationships that could be construed as a potential conflict of interest.

## Abbreviations

ALL: acute lymphoblastic leukemia
AR^-^: hormone-refractory
AR^+^: hormone-sensitive
ASCL1: achaete scute complex like 1
CCA: cholangiocarcinoma
EGCG: epigallocatechin-3-gallate
EGFR: epidermal growth factor receptor
HCC: hepatocellular carcinoma
HPV: human papillomavirus
MCF-7/ADR: adriamycin-resistant MCF-7
MDR1: multi-drug resistance 1
NSCLC: non-small cell lung cancer
PCa: prostate cancer
STAT3: signal transducer and activator of transcription 3
TRAIL: tumor necrosis factor-related apoptosis-inducing ligand
TRAMP: transgenic adenocarcinoma of the mouse prostate
Xn: xanthohumol
